# Analysis of Growth Velocity in Children with Attention-Deficit/Hyperactivity Disorder Treated for up to 12 Months with Serdexmethylphenidate/Dexmethylphenidate

**DOI:** 10.1089/cap.2023.0012

**Published:** 2023-05-17

**Authors:** Ann C. Childress, Andrew J. Cutler, Maitrey Patel, Charles Oh

**Affiliations:** ^1^ Center for Psychiatry and Behavioral Medicine, Las Vegas, Nevada, USA.; ^2^ SUNY Upstate Medical University, Syracuse, New York, USA.; ^3^ Neuroscience Education Institute, Lakewood Ranch, Florida, USA.; ^4^ Corium, LLC, Boston, Massachusetts, USA.

**Keywords:** ADHD, Azstarys, SDX/d-MPH, growth velocity, *Z*-score

## Abstract

**Objective:**

Serdexmethylphenidate/dexmethylphenidate (SDX/d-MPH) is approved for the treatment of patients aged ≥6 years with attention-deficit/hyperactivity disorder (ADHD). A 12-month, open-label safety study with SDX/d-MPH in children with ADHD showed that SDX/d-MPH was well tolerated and comparable with other methylphenidate products. In this *post hoc* analysis of the 12-month study, the objective was to characterize the effect of SDX/d-MPH on growth in children with ADHD over 12 months.

**Methods:**

This was a *post hoc* analysis of a dose-optimized, open-label, phase 3 safety study of SDX/d-MPH in children aged 6–12 years with ADHD (NCT03460652). Weight and height *Z*-score analyses were conducted. *Z*-score change from baseline was calculated based on the baseline values for the subjects remaining in the study at the observation time point.

**Results:**

Subjects (*N* = 238) from the treatment-phase safety population included all enrolled subjects who received ≥1 dose of study drug and had ≥1 postdose safety assessment. During treatment, the mean weight and height *Z*-scores decreased over time from their respective baselines. At the 12-month time point, mean (standard deviation [SD]) *Z*-score changes from baseline for weight and height for the subjects remaining in the study were −0.20 (0.50) and −0.21 (0.39), respectively; however, these mean changes in Z-scores were not clinically significant (change <0.5 SD). Long-term treatment with SDX/d-MPH was associated with modest reductions in expected weight and lower-than-expected increases in height: effects that plateaued or diminished later in treatment.

**Conclusion:**

The overall effects of SDX/d-MPH on growth velocity (the change in weight and height from one time point to the next) were minimal, and the range of changes was not considered clinically significant. ClinicalTrials.gov identifier: NCT03460652.

## Introduction

Methylphenidate (MPH) is a commonly prescribed medication for attention-deficit/hyperactivity disorder (ADHD) because of its efficacy for symptom reduction and overall favorable safety record. MPH is often prescribed for long-term use in patients with ADHD (Storebø et al. 2018; Krinzinger et al. 2019). Serdexmethylphenidate (SDX) is a prodrug of dexmethylphenidate (d-MPH) and is coformulated with immediate release d-MPH in fixed molar dose ratios of 70% SDX:30% immediate release d-MPH hydrochloride (HCl).

This formulation of SDX/d-MPH (Azstarys™; Corium, LLC, Boston, MA) was approved by the US Food and Drug Administration for patients aged 6 years and older with ADHD.

In a pivotal, randomized, placebo-controlled, double-blind laboratory classroom study of children with ADHD, SDX/d-MPH treatment was shown to significantly improve ADHD symptoms compared with placebo (Kollins et al. 2021). SDX/d-MPH onset began by 30 minutes postdose, and the treatment effect lasted up to 13 hours. Adverse events (AEs) were similar to other stimulants used for the treatment of patients with ADHD.

A subsequent, 1-year, open-label safety study of SDX/d-MPH in children with ADHD showed that SDX/d-MPH was well tolerated and had sustained efficacy during the 1-year treatment period (Childress et al. 2023). Of the 238 subjects assessed in the 1-year study, the most common treatment-emergent AEs were decreased appetite (18.5%), upper respiratory tract infection (9.7%), nasopharyngitis (8.0%), decreased weight (7.6%), and irritability (6.7%).

Long-term treatment with stimulants, such as MPH for ADHD, in children has been associated with modest slowing of growth velocity and prescribing information for these treatments warns of long-term growth suppression, which can cause concern and limit their use (Spencer et al. 2006; Faraone and Giefer 2007; Krinzinger et al. 2019). The objective of the current investigation was to characterize the effects of SDX/d-MPH on growth in children with ADHD treated with SDX/d-MPH from the 1 year, open-label, safety study (Childress et al. 2023).

## Methods

This *post hoc* analysis of a dose-optimized, open-label, phase 3 safety study of SDX/d-MPH in children aged 6–12 years with ADHD (NCT03460652) (Childress et al. 2023) was conducted at 18 sites in the United States. The original study protocol and amendments were approved by an institutional review board before each center initiated the study. The first patient was screened on February 21, 2018, and the last follow-up visit was on June 27, 2019.

### Subjects

The subjects were children aged 6–12 years with ADHD at the start of the dose-optimization (DO) phase of the study. Subjects included those rolled over from the antecedent pivotal study (Kollins et al. 2021) within 45 days of their last dose of SDX/d-MPH, and new subjects who did not participate in the pivotal study or who entered the trial more than 45 days after their last dose in the pivotal study (Childress et al. 2023). Subjects had to have a body weight of at least 21 kg at the screening phase and be in good health. At least one parent or legal guardian was required to provide written permission, and each subject had to give written or verbal permission for study participation.

### Study design

The study design included a 30-day screening phase, a 3-week DO phase, a 360-day treatment phase, and a follow-up visit. Only new subjects underwent screening and DO. During the DO phase, new subjects were titrated to their optimized dose based on the best dose response and individual tolerability to the treatment (Childress et al. 2023).

New subjects started treatment with the 39.2/7.8 mg dose of SDX/d-MPH daily, which is a molar equivalent to 30 mg of total d-MPH HCl, for 7 days. If needed, dose adjustments were performed based on the investigator's assessment of the subject at weekly intervals. The SDX/d-MPH dose could be increased to 52.3/10.4 mg (40 mg molar equivalent of total d-MPH HCl), decreased to 26.1/5.2 mg (20 mg molar equivalent of total d-MPH HCl), or maintained at the same dose for the next week of dosing.

The dose at the end of the third week was assigned as the optimized dose. Subjects who rolled over from the pivotal study remained on their optimized dose from that study. The optimized SDX/d-MPH dose was used during the treatment phase. All subjects were administered 1 capsule daily of their optimized dose of SDX/d-MPH. Weight and height analyses were conducted in the treatment phase safety population of enrolled subjects. This population included those who received at least 1 dose of study medication and had at least 1 postdose safety assessment in the treatment phase. Completers are those subjects that completed 12 months of treatment.

### Weight and height measurements

Body weight (in kg) and height (in cm) were measured at each monthly visit. Body weight was measured using a calibrated scale while subjects remained in their normal clothing, with shoes and jacket (and/or outer clothing) removed. Height was measured using a stadiometer with the subject's shoes removed.

### Assessments

The predicted weight and height for each subject and visit were calculated based on the United States 2000 Centers for Disease Control (CDC) Growth Charts (ages 2 to <20 years) (Kuczmarski et al. 2002), using each subject's observed baseline weight or height. The predicted weight and height assumed maintenance of baseline weight and height percentile for each subject. The predicted weight and height over time were used as references to assess the trajectory of the observed weight and height over time.

*Z*-score, which expresses an anthropometric value as the number of standard deviations (SDs) below or above the mean of a reference population, was calculated for each subject at each visit. The reference population was based on the United States 2000 CDC Growth Charts (ages 2 to <20 years) (Kuczmarski et al. 2002). *Z*-scores were assessed against the reference population for both weight and height (Kuczmarski et al. 2002), as follows:

$$Z-score=\frac{observedvalue-meanofthereferencepopulation}{SDofthereferencepopulation}$$

A *Z*-score change <0.5 SD is considered not clinically significant. *Z*-score change from baseline was calculated based on the baseline values for the subjects remaining in the study at the observation time point. Percentiles were assessed as the percentage of observations that fell below a certain value for the reference population. *Z*-score by baseline body mass index (BMI) quartile was calculated based on the following baseline BMI quartiles: quartile 1, BMI ≤16.1; quartile 2, BMI >16.1 to ≤17.8; quartile 3, BMI >17.8 to ≤21.6; quartile 4, BMI >21.6.

The descriptive statistics included number of subjects (*n*), mean, SD, standard error, and *Z*-score. No statistical tests were conducted.

## Results

### Subject disposition

**FIG. 1.**
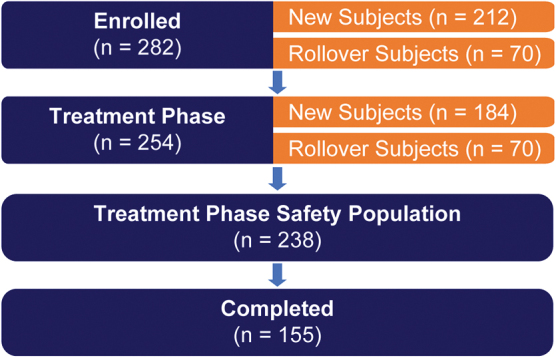
Subject disposition.

Subject disposition has been previously described (Childress et al. 2023). Briefly, 323 subjects were screened, among whom 282 were enrolled in the study. Of the 282 enrolled subjects, 254 entered the treatment phase (which included 70 rollover subjects and 184 new subjects), of whom 238 subjects were included in the treatment phase safety population (Fig. 1).

### Subject demographics and baseline characteristics

The mean (SD) age for the subjects was 9.1 (1.9) years; 60.9% of the subjects were male, and 39.1% were female. The mean (SD; minimum, maximum) for weight was 38.6 (13.9; 21.0, 97.0) kg, BMI was 19.2 (4.6; 13.1, 37.5) kg/m^2^, and height was 139.6 (11.9; 113.5, 171.2) cm (Table 1). Additional subject characteristics have been previously reported (Childress et al. 2023).

**Table 1.** Subjects' Demographics. Treatment Phase Safety Population

Parameter	Total (*N* = 238)
Age, years	
Mean (SD)	9.1 (1.87)
Minimum, maximum	6, 12
Sex, *n* (%)	
Male	145 (60.92)
Female	93 (39.08)
Weight, kg	
Mean (SD)	38.6 (13.9)
Minimum, maximum	21.0, 97.0
Height, cm	
Mean (SD)	139.6 (11.92)
Minimum, maximum	113.5, 171.2
Body mass index, kg/m^2^	
Mean (SD)	19.2 (4.6)
Minimum, maximum	13.1, 37.5

SD, standard deviation.

### Growth velocity

#### Weight

**FIG. 2.**
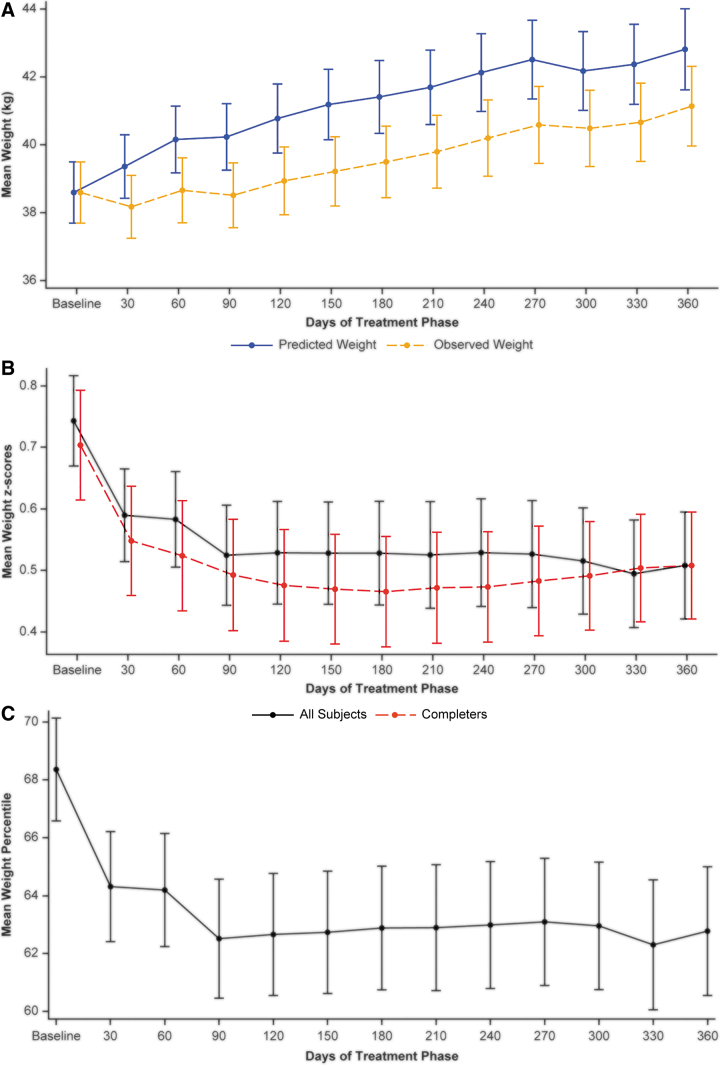
Mean observed body weight versus mean predicted body weight **(A)**, mean weight *Z*-score by completion status **(B)**, and mean body weight percentile **(C)**. Treatment phase, safety population. All subjects: *n* = 238. Completers: *n* = 155. The black curve represents all subjects remaining in the study at each visit (completers + drop-outs before they dropped out). The red curve represents completers who are subjects that completed 12 months of treatment. Bars are standard error.

The mean (SD) body weight at baseline was 38.6 (13.9) kg with a *Z*-score of 0.74 (1.13) (Table 2), indicating a baseline weight higher than the mean expected body weight for the reference population. In the first 30 days after initiating the treatment phase, mean observed weight decreased slightly, and this was followed by 2 months of slowdown in observed weight gain (Fig. 2A). After this slowdown, a steady increase in observed body weight paralleled the predicted weight curve, but at a lower starting point because of the weight deficits in the study population during the first 3 months.

**Table 2.** Baseline Observed Mean Body Weight and Height, Predicted Weight and Height, Weight and Height Z-Score, and Weight and Height Percentiles: Treatment Phase Safety Population

Parameter, mean (SD)	Weight (kg),* n = *238	Height (cm),* n = *238
Observed	38.6 (13.9)	139.6 (11.9)
Predicted	38.6 (13.9)	139.6 (11.9)
Z-score	0.74 (1.13)	0.54 (0.98)
Percentile	68.4 (27.4)	64.9 (26.6)

SD, standard deviation.

At 90 days, the mean observed weight and predicted weight were 38.5 and 40.2 kg, respectively, with a difference between the means of 1.7 kg. At the end of the study (360 days), the mean observed weight and predicted weight were 41.1 and 42.8 kg, respectively, with the same difference between the means of 1.7 kg as after 3 months of treatment.

The mean (SD) weight *Z*-score at baseline was 0.74 (1.13). Mean weight *Z-*score decreased during the first month in the treatment phase, as evidenced by the decline at 30 days (Fig. 2B). Most of the mean weight Z-score decline occurred in the first 4 months of treatment and remained stable thereafter, near 0.5. For all subjects, the mean (SD) weight *Z*-score during the first 3 months was 0.53 (1.17), and at 12 months, it was 0.51 (1.08), a change from baseline of −0.22 (0.31) at 3 months and −0.20 (0.50) at 12 months based on the baseline scores for those subjects remaining in the study at 3 and 12 months, respectively.

For subjects who completed the study, smaller decreases or lower-than-expected increases were observed during the second and third months, after which the weight velocity resumed, although at a lower starting weight at 90 days. The average weight decreased from the 68.4 (27.4) percentile to the 64.3 (28.8) percentile during the first month in the treatment phase, with an additional small decrease to the 62.8 (27.7) percentile by 12 months (Fig. 2C).

Approximately the same mean weight percentile was maintained for the remainder of the study. The percentage of subjects with clinically notable weight loss, defined as a weight decrease from baseline >7%, was 8.7% at 30 days. The percentage of subjects with weight loss increased over time, peaking at 14.5% of subjects at 4 months and then declining to 5.2% of subjects at the end of the study (360 days). This time course is consistent with average weight loss observed during the beginning of the treatment phase followed by weight gains later in the study.

**FIG. 3.**
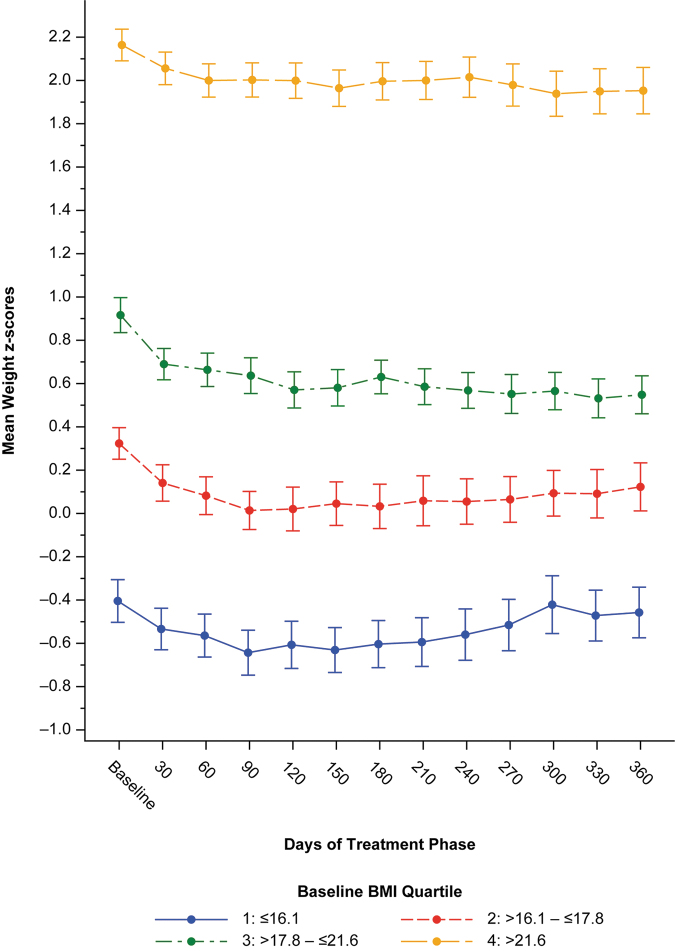
Mean weight *Z*-score by baseline BMI quartile. Treatment phase, safety population. Bars are standard error. BMI, body mass index.

The mean weight *Z*-scores over time by baseline BMI quartiles are shown in Figure 3. The mean baseline Z-scores were −0.40, 0.32, 0.92, and 2.16, for baseline BMI quartiles 1, 2, 3, and 4, respectively. At the end of treatment, the mean Z-scores were −0.46, 0.12, 0.55, and 1.95, a change from baseline of −0.07, −0.13, −0.33, and −0.25 for baseline BMI quartiles 1, 2, 3, and 4, respectively.

The Z-scores for subjects with a baseline BMI <16.1 kg/m^2^ approached the baseline *Z*-score by the end of the study after a decrease in weight *Z*-score during the initial 3 months of treatment. The weight *Z*-score of subjects with mean BMI >21.6 kg/m^2^ at baseline decreased during the initial 2 months of treatment and remained approximately constant thereafter.

#### Height

The mean (SD) height at baseline was 139.6 (11.9) cm with a *Z*-score of 0.54 (0.98) (Table 2), indicating that the baseline height was higher than the mean expected body height for the reference population (i.e., the subjects in the present study were taller than the expected height for their age, according to CDC norms).

**FIG. 4.**
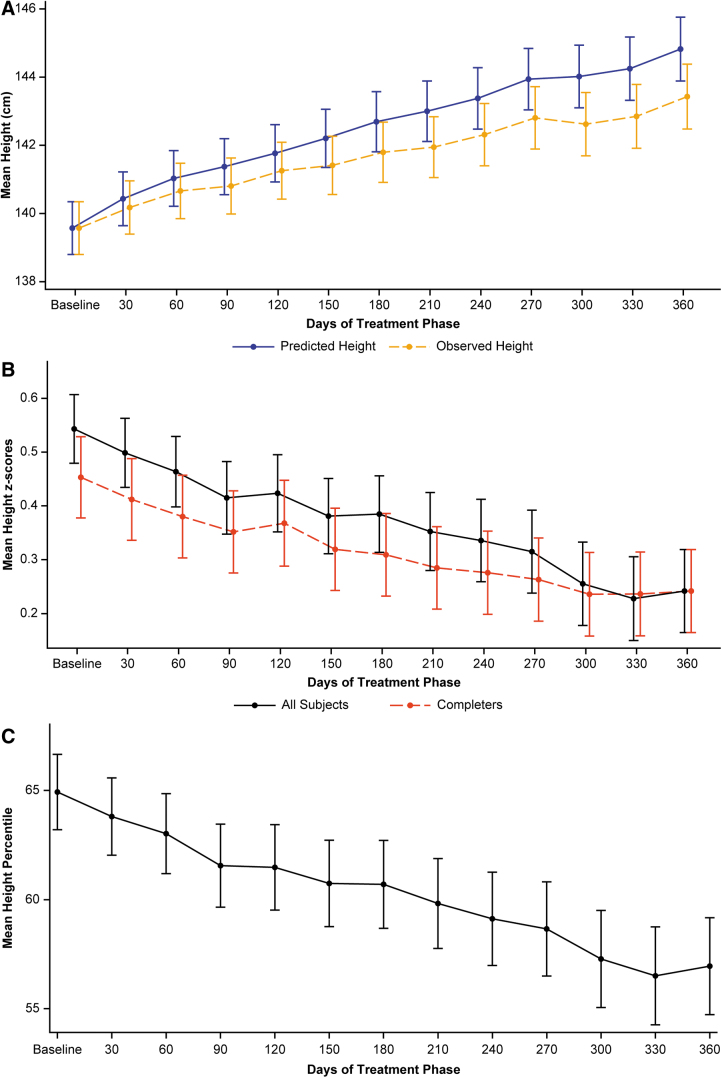
Mean observed height versus mean predicted height **(A)**, mean height Z-score by completion status **(B)**, and mean height percentile **(C)**. Treatment phase, safety population. All subjects: *n* = 238. Completers: *n* = 155. The black curve represents all subjects remaining in the study at each visit (completers + drop-outs before they dropped out). The red curve represents completers who are subjects that completed 12 months of treatment. Bars are standard error.

A comparison of observed height versus predicted height showed a steady increase of the mean observed height over time, which was at a lower rate than the predicted velocity of height gain at each monthly visit (Fig. 4A). In the treatment phase at 90 days, the mean observed height and predicted height were 140.8 and 141.4 cm, respectively, with a difference between the means of 0.6 cm. At 180 days, the mean observed height and predicted height were 141.8 and 142.7 cm, respectively, with a difference between the means of 0.9 cm. At the end of the study, the mean observed height and predicted height were 143.4 and 144.8 cm, respectively, with a difference between the means of 1.4 cm.

Mean height *Z*-scores decreased over time (Fig. 4B). The mean (SD) baseline height *Z*-score for all subjects was 0.54 (0.98). The greatest mean Z-score decrease occurred at 6 months. The mean height *Z*-score decreased to 0.39 (0.98) after 6 months in the treatment phase, a change from baseline of −0.14 (0.28), and decreased to 0.24 (0.96) after 12 months, a change from baseline of −0.21 (0.39), based on the baseline scores for those subjects remaining in the study at 6 and 12 months, respectively.

Mirroring the mean height Z-score, the mean height percentile steadily decreased, with the mean (SD) height percentile decreasing from the 64.9 (26.6) to the 60.7 (27.7) percentile after 6 months in the treatment phase and to the 57.0 (27.7) percentile at 12 months (Fig. 4C).

**FIG. 5.**
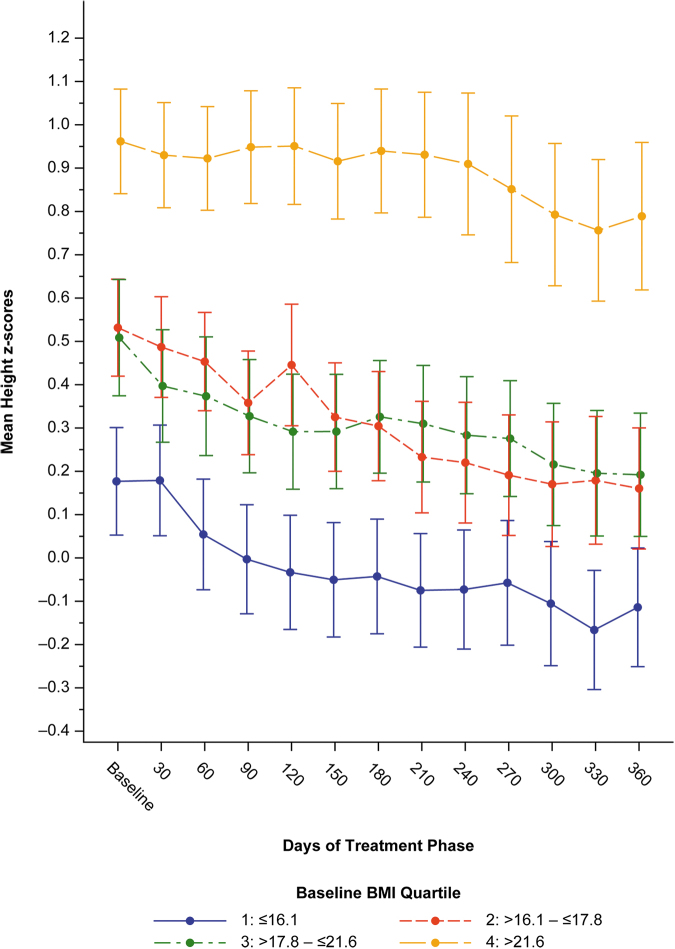
Mean height *Z*-score by baseline BMI quartile. Treatment phase, safety population. Bars are standard error. BMI, body mass index.

The mean baseline height Z-scores were 0.18, 0.53, 0.51, and 0.96, for baseline BMI quartiles 1, 2, 3, and 4, respectively (Fig. 5). At the end of treatment, the mean *Z*-scores were −0.11, 0.16, 0.19, and 0.79, a change from baseline of −0.25, −0.25, −0.23, and −0.04, for baseline BMI quartiles 1, 2, 3, and 4, respectively. The mean height *Z*-score decrease from baseline was much lower in the baseline BMI quartile 4 (which also had a higher mean baseline height *Z*-score) than in the lower 3 baseline BMI quartiles.

## Discussion

The safety and tolerability results from this 1-year open-label study of SDX/d-MPH in children with ADHD have been previously reported (Childress et al. 2023). Decreased weight was reported as a treatment-emergent AE, occurring in 7.6% of subjects. In this analysis, baseline mean weight and height were higher in study subjects than in the reference population.

Other studies of MPH in children with ADHD also observed similarly higher baseline weight and height than their reference samples (Swanson et al. 2006; Childress et al. 2022). In this analysis, we showed a transient decrease in observed body weight followed by a slower-than-predicted weight gain in the first 3 months of SDX/d-MPH treatment. Subsequently, the resulting difference between observed and predicted mean body weights remained fairly constant to the end of the treatment phase. In the present study, the change in weight Z-score from baseline to the study's end at 12 months was −0.20.

A *Z*-score change <0.5 SD is considered not clinically significant. The mean weight *Z*-score decreased initially, with the largest mean Z-score decrease occurring during the first month of the treatment phase, with smaller decreases in the second and third month. These results are consistent with published data, suggesting that the rate of accumulating weight deficits is attenuated over time with continued stimulant treatment (Faraone et al. 2008).

The mean weight changes as a percent from baseline-by-baseline BMI quartile groups showed that all four baseline BMI quartile groups had an initial weight decrease during the first to fourth months in the treatment phase, with a notable smaller and shorter weight decrease in baseline BMI quartile 1 compared with the other three baseline BMI quartile groups.

The relative weight gain after the initial decline through the end of treatment was higher in subjects in the first and second baseline BMI quartile groups compared with subjects in the third and fourth baseline BMI quartile groups. This difference in weight gain indicates that subjects with a higher baseline BMI experienced a larger initial reduction in weight gain than subjects with a lower baseline BMI. Relative weight gain after ∼3 months of treatment appeared similar between all baseline BMI quartiles.

With respect to height, the positive mean baseline height *Z*-score of 0.54 for all study subjects indicates that the average baseline height was higher than the mean expected height for the reference population. Mean height increased steadily during the study, although the height increases were lower when compared against the reference population. Mean height *Z*-scores and height percentiles decreased over time.

The change in height *Z*-score from baseline to the end of the study was −0.21. We also showed that the mean height *Z*-score decrease from baseline was much lower in the upper baseline BMI quartile group (−0.04 for fourth baseline BMI quartile) than in the lower three baseline BMI quartiles (−0.25, −0.25, and −0.23 for first, second, and third baseline BMI quartile groups, respectively).

The effects of long-term treatment of stimulants on weight and height in pediatric patients are well documented (Spencer et al. 2006; Swanson et al. 2006; Faraone et al. 2008, 2010). A 5-week study of 253 children aged 6–12 years with ADHD receiving daily doses of extended release d-MPH found that the percentage of subjects with clinically notable weight loss (a weight decrease from baseline >7%) was 5.8%, 4.0%, and 7.7%, after daily doses of 10, 20, and 30 mg extended release d-MPH (vs. 0% after placebo) (Childress et al. 2009).

However, that study was of a much shorter span than the current study, consisting of a 3-week dose optimization phase followed by a 2-week maintenance phase, and it is possible that study had not reached maximum effect on body weight that may have been measured later in treatment had the treatment continued.

In a 21-month study with a dose-optimized long-acting MPH formulation in 407 children aged 6–13 years with ADHD (Spencer et al. 2006), the mean baseline weight *Z*-score was higher than 0, indicating that the children in that study were on average heavier than the CDC reference sample. In that study, the largest weight Z-score decrease occurred during the first 5 months of treatment (decreased from 0.16 to −0.11) and then remained relatively stable for the remainder of the study.

In a more recent 12-month open-label safety study of extended-release MPH in children aged 4–5 years with ADHD, subjects entering the study were heavier and taller than the reference population (Childress et al. 2022). Weight decrease was a commonly reported treatment-emergent AE (Childress et al. 2022), as was found in the 1-year safety study of SDX/d-MPH (Childress et al. 2023).

In the extended-release MPH study (Childress et al. 2022), decrease in weight-for-age percentiles was smallest for subjects in the lowest quartile of baseline weight (−8.1) versus the other three baseline weight quartiles (−14.0, −10.0, and −11.4 for second, third, and fourth quartiles, respectively). In addition, the smallest changes in height percentiles were seen for children in the first quartile of baseline height (−2.4), compared with the second (−10.0), third (−8.7), and fourth (−9.7) baseline height quartiles.

The limitations of this study include the open-label nature of the study design and the lack of a control placebo arm or a comparator product. The analysis of a drug effect on body weight changes over time by itself is limited because children are expected to grow and gain weight as they age, even during SDX/d-MPH treatment. In addition, some of the subjects who entered this study were not stimulant naive, as some had been previously treated with SDX/d-MPH in the antecedent pivotal study. Thus, previous stimulant exposure may have impacted baseline height and weight measures, resulting in an underestimate of growth velocity changes.

## Conclusion

In this study, we show that long-term treatment for up to 1 year with SDX/d-MPH was associated with modest reductions in expected weight and lower-than-expected increases in height, effects that plateaued or diminished later in treatment. Although both weight and height decreased compared with the reference population, the decrease was less than what is clinically significant.

## Clinical Significance

This phase 3 clinical trial of SDX/d-MPH in children aged 6–12 years showed that the overall effects of SDX/d-MPH on growth velocity were minimal and changes were in a range considered not clinically significant.
